# Record of polycyclic aromatic hydrocarbons (PAHs) from prehistoric sediments and human activity in the Lubei plain of China

**DOI:** 10.1038/s41598-025-91885-1

**Published:** 2025-03-01

**Authors:** Huanrong Zuo, Zhihai Tan, Yongming M Han, Longjiang Mao, Shuxin Zheng, Qi Zhang, Meng Wang, Shihao Li

**Affiliations:** 1 School of Environment and Chemistry Engineering, Xi’an Polytechnic University, Xi’an, 710048 Shaanxi China; 2 State Key Laboratory of Loess and Quaternary Geology, Key Laboratory of Aerosol Chemistry and Physics, Institute of Earth Environment, Chinese Academy of Sciences, Xi’an, 710061 Shaanxi China; 3 School of Marine Sciences, Nanjing University of Information Science & Technology, Nanjing, 210044 Jiangsu China

**Keywords:** Polycyclic aromatic hydrocarbons (PAHs), Black carbon (BC), Anthropogenic biomass burning, Warfare, Fire pattern, Climate change, Palaeoclimate

## Abstract

Understanding the sources and dynamics of past biomass burning remain a significant challenge due to variations in paleofire combustion patterns across different temporal and spatial scales. This study integrates black carbon and polycyclic aromatic hydrocarbon (PAH) records from the Lubei Plain in the Shandong Peninsula, Lower Yellow River, to reconstruct Holocene fire regimes and their relationship with climatic shifts and human activities over the past 5000 years. During the mid-to-late Holocene (5000–3000 year BP. (calendar years before 1950)), a biomass burning levels were generally low, with a pronounced peak in low-molecular-weight PAHs (3-ring PAHs) and charcoal fluxes between 5000 and 4500 year BP, indicating increased fire activity likely driven by a short-term cold-dry event around 5000 year BP. From 3500 to 1000 year BP, three distinct episodes of low-temperature smoldering fires are identified, coinciding with deforestation and persistent droughts during the Shang Dynasty (3600–3046 year BP), the Qin and Western Han Dynasties (2200–2000 year BP), and the Sui and Tang Dynasties (1400–1100 year BP). In contrast, high-temperature flaming fires are associated with periods of intensified warfare and social upheaval, compounded by cold, arid climates during the Warring States period (2500–2400 year BP), the Eastern Han Dynasty (2000–1800 year BP), and the Wei, Jin, and Southern-Northern Dynasties (1800–1400 year BP). Over the past millennium, anthropogenic biomass burning remained elevated, reflecting sustained human influence on fire regimes. Meanwhile, Pollen and n-alkane records reveal a transition from primary forests to secondary shrubland during the late Holocene, driven by declining seasonal precipitation linked to a weakening East Asian monsoon and increased anthropogenic burning. Principal component analysis indicates that long-term deforestation primarily drove low-temperature smoldering fires, whereas high-temperature fires were more closely linked to periods of conflict. Seasonal precipitation variability, regulated by monsoonal dynamics, emerged as a fundamental control on fire regimes. This integrated analysis of PAHs, black carbon, and charcoal, coupled with multivariate statistical approaches, offers a robust framework for reconstructing fire-climate-human interactions in East Asia. The findings provide new insights into the mechanisms driving fire regimes and their long-term ecological and societal impacts.

**Supplementary Information:**

The online version contains supplementary material available at 10.1038/s41598-025-91885-1.

## Introduction

Fire is a fundamental disturbance factor in terrestrial ecosystems^1,2^. Fire regimes are typically driven by long-term climatic changes, which subsequently affect global carbon pools and biogeochemical cycles. Meanwhile fires also indirectly influences the probability of future fire events through their impact on vegetation evolution^2,3^. Since prehistoric times, human activities involving various biomass burning practices have significantly altered and will continue to modify evolution of the natural landscape^4–6^. Reconstructing ancient fire histories has become an indispensable method for elucidating the processes of natural landscape evolution throughout geological history.

Charcoal and black carbon (BC) preserved in sediments and loess are widely recognized as common proxies for reconstructing ancient fire histories^7,8^. Variations in charcoal concentration provide insights into past fire intensity, frequency, and burned area^4,9,10^. Macro-charcoal particles (> 125 μm) are generally indicative of local fire events^11,12^, whereas micro-charcoal particles (< 50 μm) reflect regional fire activity^13^. The chemical properties of charcoal are inert, with combustion temperatures ranging from 250 °C to 500 °C^7^. Charcoal forms a part of the black carbon (BC) continuum^14^. Black carbon, primarily derived from the incomplete biomass combustion, consists of carbon-rich compounds, including char and soot^7,8,15–17^. Char particles (with a particle size > 1 μm) serve as indicators of regional wildfire activity^18–20^. These particles are formed at combustion temperatures exceeding 350 °C, typically under smoldering conditions in relatively humid environments^7,16,17^. Soot particles (with a particle size < 1 μm) can reflect biomass burning on a continental scale^21^ and are formed at combustion temperatures exceeding 700 °C^7,22^. Soot production typically occurs under flaming combustion conditions in relatively arid environments^7,16,17^.

Currently, a growing number of scientists recognize that analyzing charcoal and black carbon records in lakes, peat bogs, and soil sediments can significantly enhance our understanding of climate-fire-vegetation interactions and the impact of human activities on fire occurrences across different temporal and spatial scales^5,17,23–28^. Previous studies have shown that during the early Holocene on the Loess Plateau, there was a notable peak in charcoal concentrations, whereas char peaks were less pronounced. These findings suggest that fire occurrences were closely linked to the type of vegetation (fuel characteristics) and combustion temperatures^5^. More recently, a detailed fire history from the past 5,000 years was reconstructed in the Changyi region of Shandong Province, China. During the periods of 5300–5100 year BP and 4400–3600 year BP, under relatively humid conditions, Char concentrations attained peaks, while charcoal levels remained low. In contrast, Char concentrations were low, whereas charcoal concentrations reached a peak between 3600 and 3200 year BP with relatively arid conditions^29^. These differences suggest that fire occurrences are likely influenced by variations in combustion temperatures and depositional environments, which in turn affect fuel characteristics and fire behavior (e.g., smoldering vs. flaming). These variations lead to distinct physical and chemical emissions from combustion^30^. In relatively humid environments, low-temperature smoldering fires are more common, though they may be underestimated in paleofire reconstructions when charcoal is used as a proxy due to their lower visibility in the charcoal record. Traditional fire reconstruction methods often emphasize fire frequency and intensity, while overlooking the origins of fires, particularly those driven by human activities such as agriculture, warfare, and domestic practices. Relying solely on variations in the concentrations of these proxies may bias our understanding of fire patterns. Therefore, it is crucial to introduce a more robust biomass combustion proxy that accurately identifies the sources of paleofires and reflects vegetation characteristics. This study aims to integrate molecular biomarkers of polycyclic aromatic hydrocarbons (PAHs) with sedimentary records of charcoal and black carbon to evaluate and determine the role of human-induced biomass burning in shaping prehistoric landscapes.

PAHs are classified as persistent organic pollutants (POPs) under the United Nations Economic Commission for Europe (UNECE) Convention. In widely distributed sediments, PAHs primarily originate from natural forest fires and volcanic eruptions^31^, as well as from anthropogenic biomass combustion emissions^32–35^. PAHs are subsequently transported to surface soils through both dry and wet deposition processes^36^. The sources of PAHs can be categorized into two main types: pyrogenic and petrogenic. Pyrogenic PAHs are derived from fossil fuels and biomass combustion, whereas petrogenic PAHs originate from crude oil and petroleum products, including kerosene, gasoline, diesel, lubricating oil, and asphalt^37–39^. Notably, pyrogenic PAHs differ from petrogenic PAHs, which are petroleum-based and often display branched or substituted structures^40^. As molecular biomarkers, PAHs offer valuable insights into fire source analysis due to their stable and persistent molecular structures^14,41–44^. They enable the rapid and accurate detection of fire-related signals, including those associated with human activities and agricultural practices^45^. Low molecular weight PAHs (2-ring PAHs), such as naphthalene, are more susceptible to microbial degradation, photochemical oxidation, and volatilization due to their higher solubility and volatility^46,47^. These characteristics, along with variations in combustion conditions, fuel sources, depositional environments, and post-depositional transformations, may limit the reliability of low molecular weight PAHs (2-ring PAHs) as indicators of past fire events. In contrast, higher molecular weight (HMW) PAHs are more hydrophobic and show greater resistance to biodegradation, particularly under the anoxic conditions typical of freshwater sediments. Recent studies have demonstrated that the vertical stratification of PAHs in sediment profiles provides valuable records of fire activity, extending over millions of years^48^.

Preliminary studies have shown that pyrogenic PAH compounds, derived from the incomplete combustion of biomass and fossil fuels^49,50^, are increasingly recognized as reliable proxies for vegetation burning in sedimentary records^14,43,51–54^. Research has demonstrated that analyzing PAHs in sediments, when integrated with other proxy indicators, can elucidate the correlations between fire history and climatic events dating back to the Late Ice Age^55–57^. It has been established that low molecular weight PAHs (3-ring PAHs), such as fluoranthene, pyrene, and benzo[a]anthracene, serve as effective biomarkers for fire events, while HMW PAHs are indicative of fire intensity^41,42^. Recent studies indicate that polycyclic hydrocarbons in sediments, as specific molecular markers, can provide detailed insights into anthropogenic activities, fire dynamics, and land use changes^14,43,44^. Previous findings indicate a significant positive correlation between PAH concentrations in Holocene sediments from the southern Loess Plateau and fire events^45^. However, the relationships between PAH components (low molecular weight and high molecular weight) and black carbon components (char and soot) in sediments remain largely unresolved.

Thus, the continental sedimentary profile of the Northwest Shandong Plain, located in the lower reaches of the Yellow River Basin, was selected as the study area. By integrating molecular biomarkers of PAHs with sedimentary records of charcoal and black carbon, this study offers a comprehensive analysis of prehistoric fire dynamics and human activities in the region.

## Regional setting and sedimentary profile

**Fig. 1:**
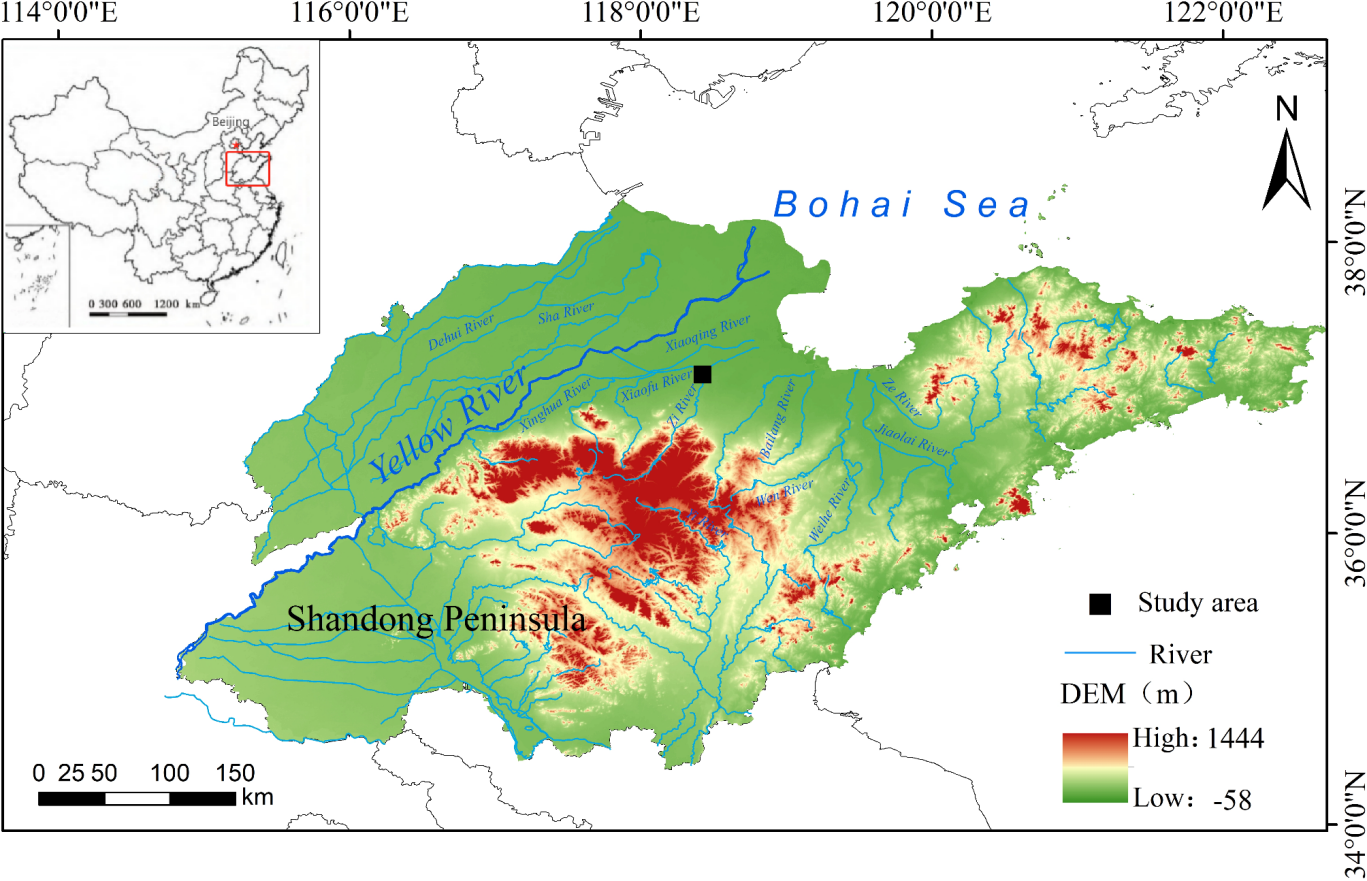
Location of the Changxu (CX) sampling point in the lower reaches of the Yellow River.

The Changxu Profile (CX) (118°25′32″E, 37°05′31″N, 13 m a.s.l.) is situated in the northwestern part of the Lubei Plain on the Shandong Peninsula, near the Bohai Sea in the lower reaches of the Yellow River (Fig. 1). This region experiences a warm temperate continental monsoon climate, primarily influenced by variations in the intensity of the East Asian monsoon. Annual precipitation ranges from 500 to 650 mm, with an average annual temperature between 12.7 °C and 14.8 °C. Winters are typically cold and dry, featuring minimal rainfall, while summers are hot and rainy, with precipitation concentrated during this season, accounting for approximately three-quarters of the annual total. These climatic conditions are frequently associated with droughts, floods, and saline-alkali disasters. The region’s vegetation primarily consists of deciduous broadleaf and temperate coniferous forests. Numerous small rivers, including the Xiaoqing River, Weihe River, and Jiaolai River, have converged over the years to form a foremountain alluvial plain. Historically, this area nurtured the Longshan Culture (4600–4000 year BP) and the Yueshi Culture (4000–3500 year BP), followed by the Shang-Zhou Culture (3600–2100 year BP)^58^. The study of this region offers an exceptional sediment archive to assess PAHs as a paleofire proxy^59–62^.

### Sampling, stratigraphy, and chronology

**Fig. 2:**
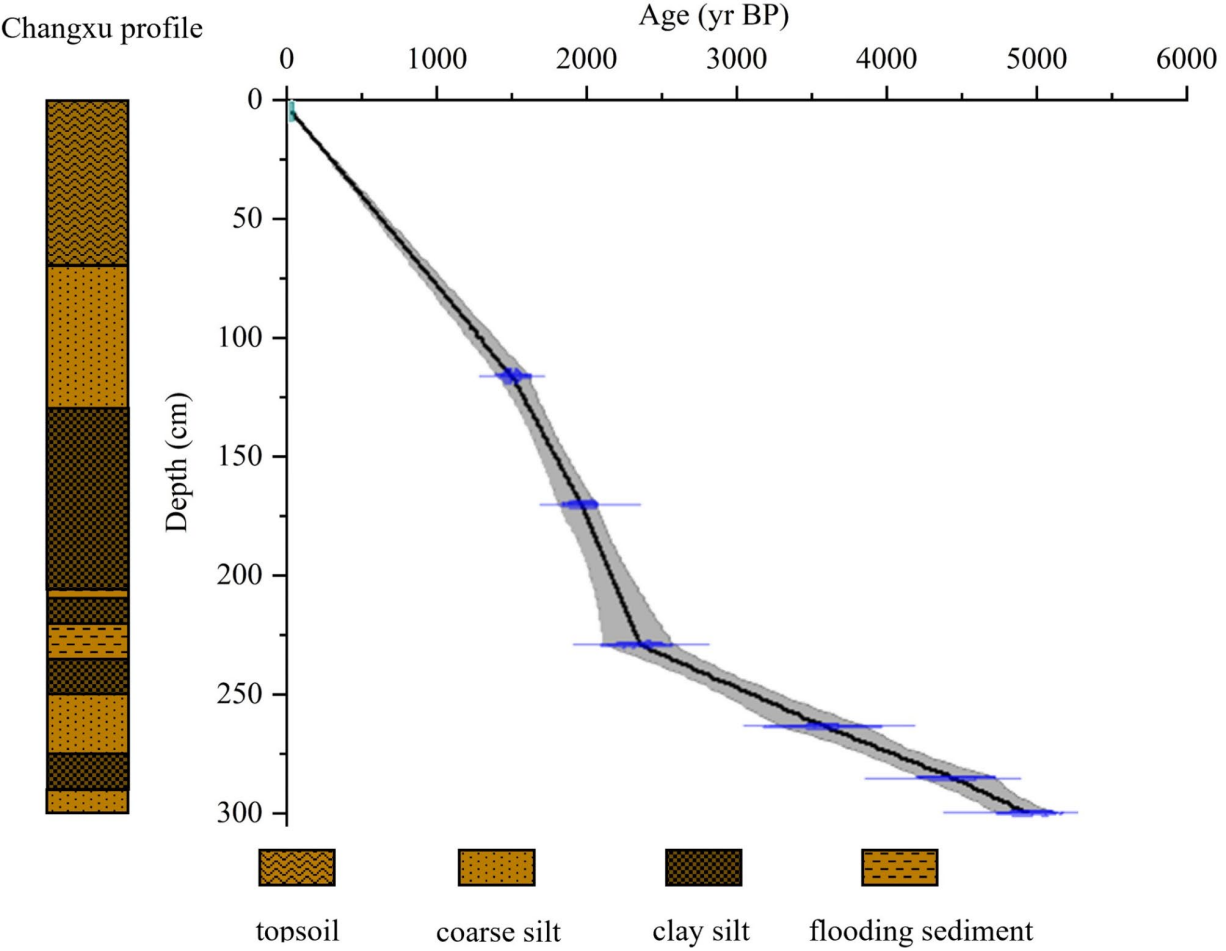
OSL dating ages versus sediment depth from the CX section in the middle and lower reaches of the Yellow River. Notably, two paleo-flood slackwater deposits were identified within the pedogenic layer at a depth of 235–205 cm.

Field investigations in the northwestern part of the Lubei Plain on the Shandong Peninsula were conducted in 2021. For this study, a Holocene terrestrial sedimentary profile at the CX site was selected (Fig. 2). The profile was sampled to a depth of 310 cm, yielding 62 samples at 5 cm intervals. Based on sedimentological characteristics such as color, texture, and structure observed in the field, the profile was divided into 10 distinct layers (Table 1). Notably, two paleo-flood slack water deposits were identified within the pedogenic layer at depths of 235–205 cm. Detailed pedo-sedimentary descriptions were performed on air-dried samples in the laboratory (Table 1).

**Table 1:** Lithological description of the CX profile.

Depth (cm)	Lithology	Stratigraphic description
0-70	Topsoil	Clumpy granular structure; a few fragments of peach pits were observed at 50 cm depth, and grey pottery shards were found between 40–50 cm depth
70-130	Coarse silt	Coarse silt, yellowish orange, the massive structure
130-155	Soil	Clayey silt, dark brownish yellow, granular structure, strong pedogenesis, many root pores and earthworm wormholes.
155-205	Clayey silty	Silty sand with a clayey texture, granular and cloddy structure, orange-yellow in color, containing a small number of wormholes and root holes.
205-206	Flooding sediments	Silt, orange, uniform and loose texture, no stratification, flood sediments.
206-220	Clayey silty	Clayey silty sand with a granular and cloddy structure.
220-235	Flooding sediments	Fine silty sand, uniform in texture, orange-yellow in color.
235-250	Clayey silty	Clayey silty sand with a granular and cloddy structure, containing a large amount of earthworm castings.
250-275	Soil	Cloddy and relatively loose.
275-290	Coarse silt	Coarse silt, yellowish orange, the massive structure
290-340	Paleosoil	Loose texture with abundant iron rust spots, relatively uniform, containing a few charcoal-colored spots; indistinct layering of mud and sand.

Optically stimulated luminescence (OSL) dating samples were analyzed at the College of Urban and Environmental Sciences, Northwest University, China (Table 2). The chronological framework for the profile was established using reliable OSL dating of sediments, archaeological artifacts, and stratigraphic divisions. The age-depth model was subsequently constructed using CLAM software, providing a chronological sequence for the study profile (Fig. 2)^63^.

**Table 2:** Optically stimulated luminescence (OSL) dating data from the CX profile.

Sample No.	Depth /cm	Particle size/μm	OSL /a BP.
CX-OSL-01	110-115	63~90	1790±40
CX-OSL-02	165-170	63~90	2220±20
CX-OSL-03	225-230	63~90	2610±50
CX-OSL-04	260-265	63~90	3.75±100

## Methods

Sediment PAHs were extracted and purified following U.S. EPA Method 3540 C and 3630 C^64,65^. Approximately 1.5 g of dry sediment was Soxhlet-extracted for 16 h using a 1:1 (v/v) acetone-dichloromethane mixture (150 mL). The extract was purified on a silica gel column (10 g), pre-washed with 40 mL of n-hexane to remove non-PAH impurities, and subsequently eluted with 20 mL of a 1:1 (v/v) methylene chloride-n-hexane mixture. The eluates were concentrated to < 0.5 mL under a nitrogen stream. PAHs were quantified using gas chromatography-mass spectrometry (GC/MS, Agilent 6890 GC-5975MS) equipped with an HP-5MS column (30 m × 0.25 mm × 0.25 μm). Helium was used as the carrier gas. The oven temperature program began at 100 °C for 1 min, ramped at 5 °C/min to 240 °C (held for 20 min), and then at 10 °C/min to 280 °C (held for 20 min). Samples (1.5 µL) were injected in splitless mode. Calibration used the external standard method with the EPA 610-N PAH Kit (Sigma Aldrich), and calibration checks were performed every 10 samples. PAH recoveries ranged from 85 to 120%.

Sediment n-alkanes were extracted and purified as described by Hu et al.^66^. Approximately 5 g of dry sediment was ultrasonicated for 30 min at 65 °C with 150 mL of a 1:1 (v/v) acetone-dichloromethane mixture. The extract was purified using a silica gel column (5 g silica gel, 2 g anhydrous sodium sulfate) eluted with 40 mL of a 1:1 (v/v) dichloromethane-n-hexane mixture. The eluates were concentrated to < 0.5 mL under nitrogen. n-Alkanes were quantified using GC/MS (Agilent 6890 N GC-5975 N MS) equipped with an HP-5 column (30 m × 0.25 mm × 0.25 μm). Helium was used as the carrier gas. The oven temperature was set at 65 °C for 5 min, ramped at 10 °C/min to 300 °C, and held for 5 min. Samples (1.5 µL) were injected in splitless mode. Concentrations of n-alkanes (n-C8 to n-C31) were calculated using external standards. Blank samples were analyzed every 10 samples, with detection limits ranging from 0.0012 to 0.0154 µg/g and recovery rates of 70.5–125.6%.

## Results and interpretation

**Fig. 3:**
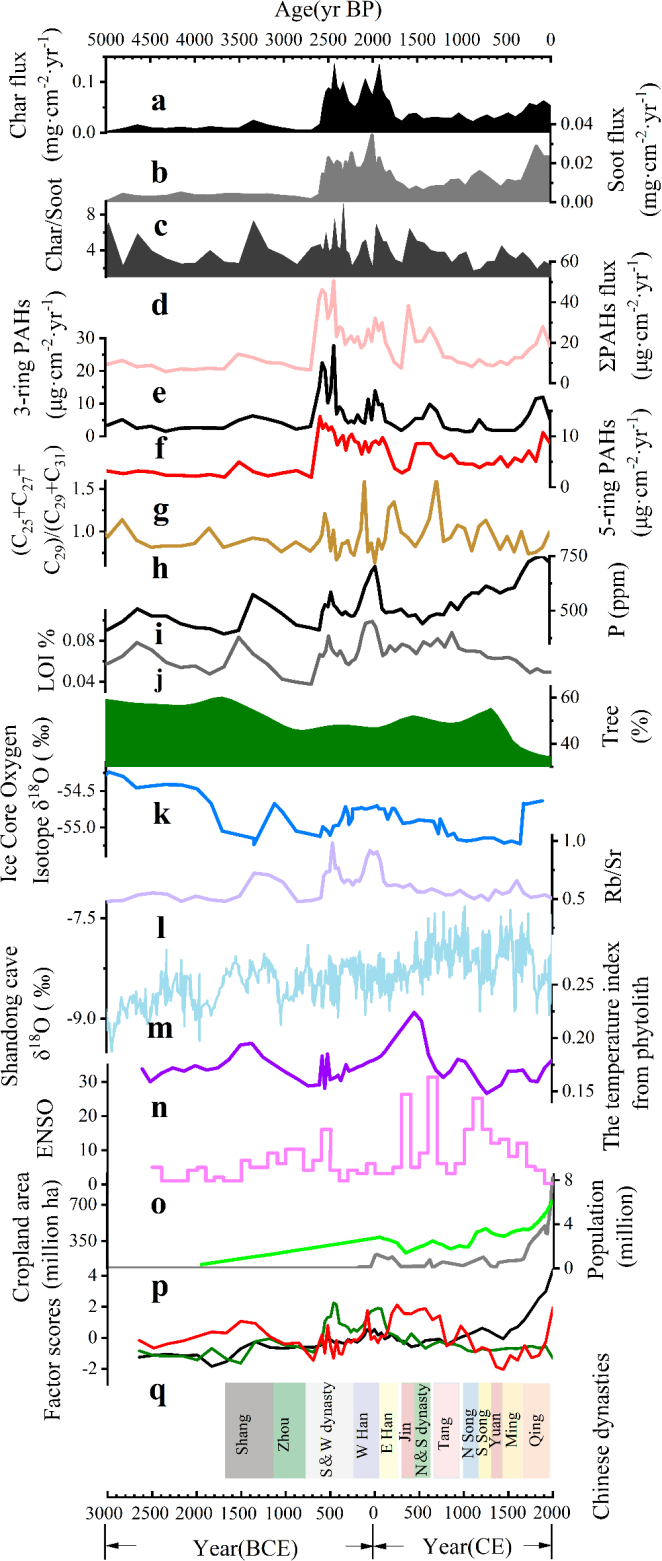
Comparison of Multi-Proxy Indicators and Fire Dynamics over the Past 5000 Years from the CX Profile. (**a**) Char flux trends from the CX site^123^; (**b**) Soot flux trends from the CX site^123^; (**c**) Ratio of char to soot concentrations from the CX site^123^; (**d**) Total PAHs (∑PAHs) flux trends from the CX site(This Study); (**e**) Trends in 3-ring PAH concentrations from the CX site(This Study); (**f**) Trends in 5-ring PAH concentrations from the CX site(This Study); (**g**) Ratio of (C25 + C27 + C29)to (C29 + C31) from the CX site(This Study); (**h**) P concentration values from the CX site^123^; (**i**) Loss on ignition (LOI) data from the CX site^123^; (**j**) Tree vegetation composition in the Bohai Sea region over the past 10,000 years^124^; (**k**) δ18O data from the Dome Fuji ice core^99^; (**l**) Ratio of Rb/Sr concentrations from the CX site^123^; (**m**) δ18O data from speleothems in Shandong Cave^98^; (**n**) Temperature index based on phytolith records from a core near Dongying Harbor^125^; (**o**) Event time series constructed using the event model, illustrating the number of events in 100-year overlapping windows^126^; (**p**) Historical population (grey line) and cropland area (green line) of the study area^10,127^; (**q**) Variations of factor score for fire with the change of depths (black color) indicating anthropogenic fires ignition; green color indicating humidity; red color indicating source of war combustion.

**Fig. 4:**
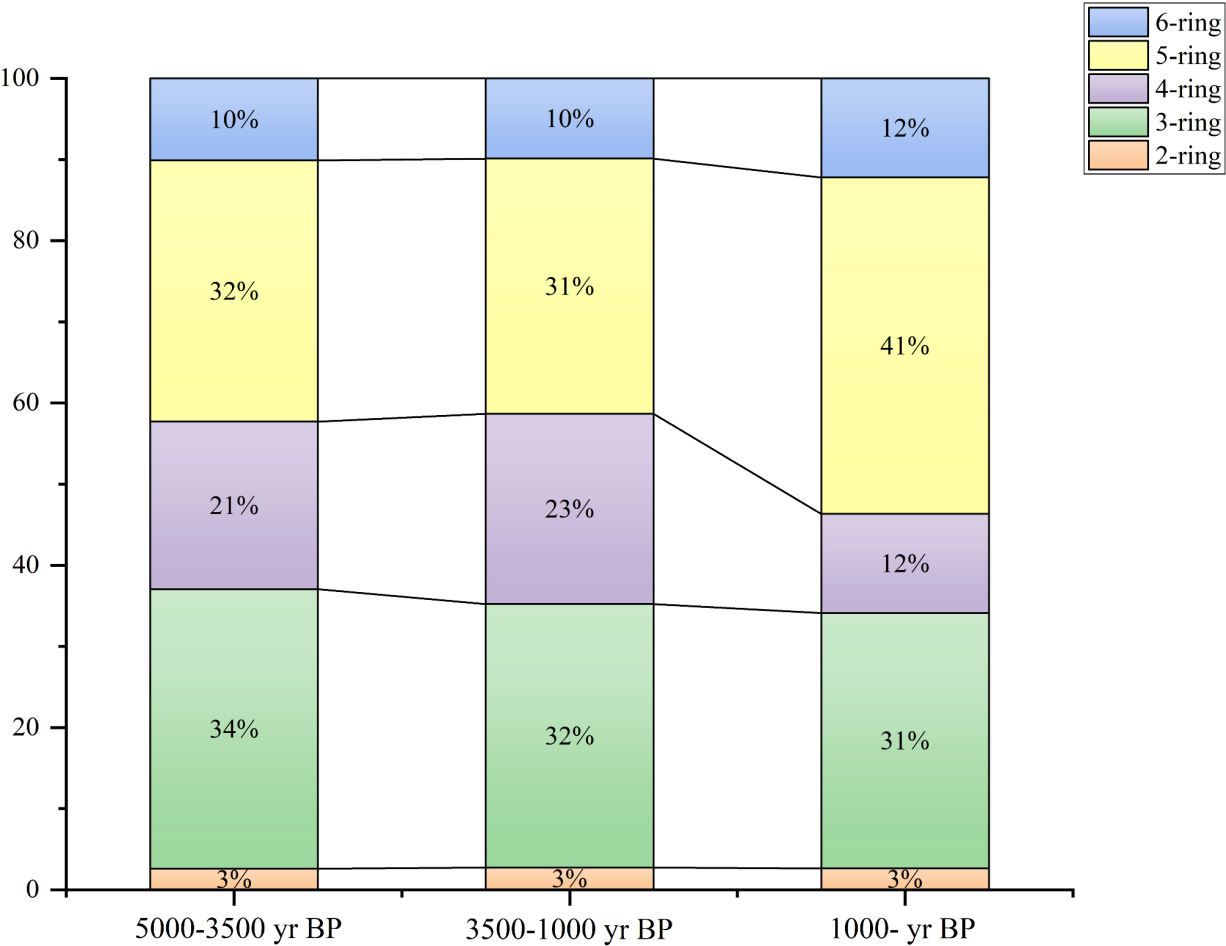
Accumulation diagram of PAH rings across three distinct time periods: 5000–3500 year BP, 3500–1000 year BP, and 1000 year BP to the present.

In the CX profile, 15 PAHs were detected, with the total PAH flux (∑PAHs) ranging from 5.74 to 50.53 µg·cm^−^²·yr⁻¹ and an average of 18.5 ± 10.92 µg·cm^−^²·yr⁻¹. Low molecular weight PAHs (3-ring PAHs) exhibited fluxes of 5.29–11.99 µg·cm^−^²·yr⁻¹ (mean: 9.57 ± 8.04 µg·cm^−^²·yr⁻¹), accounting for 31–34% of the total flux. HMW PAHs (4-ring, 5-ring, and 6-ring compounds) ranged from 8.67 to 10.72 µg·cm^−^²·yr⁻¹ (mean: 9.83 ± 2.09 µg·cm^−^²·yr⁻¹), contributing 31–41% of the total flux (Figs. 3 and 4).

Four major ∑PAHs flux peaks occurred at 3500–3000 year BP (14.37 µg·cm^−^²·yr⁻¹), 2500–2400 year BP (50.52 µg·cm^−^²·yr⁻¹), 2000–1800 year BP (32.13 µg·cm^−^²·yr⁻¹), and 1800–1400 year BP (38.38 µg·cm^−^²·yr⁻¹) (Fig. 3). These elevated fluxes suggest intensified biomass burning, likely triggered by natural events such as droughts and forest fires, as well as anthropogenic activities including deforestation and slash-and-burn agriculture^67^.

**Fig. 5:**
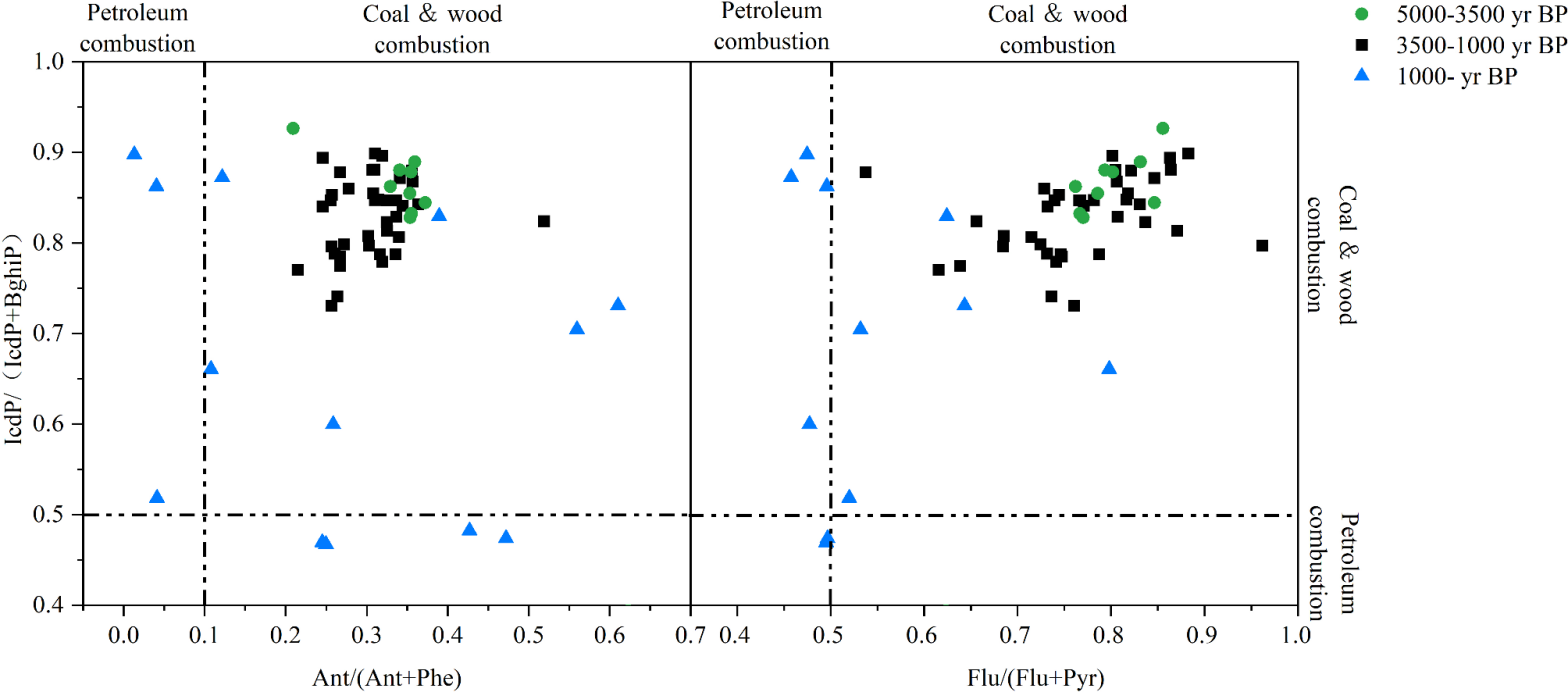
Cross-plot of polycyclic aromatic hydrocarbons (PAHs) in CX sediments. Blue triangles represent data from the last 1000 years, black squares indicate the period from 3500 to 1000 years BP, and green circles correspond to 5000 to 3500 years BP.

During various historical periods, the ratios of indeno[1,2,3-cd]pyrene/(indeno[1,2,3-cd]pyrene + benzo[ghi]perylene) (IcdP/(IcdP + Bghi)), anthracene/(phenanthrene + anthracene) (Ant/(Ant + Phe)), and fluoranthene/(fluoranthene + pyrene) (Flu /(Flu + Pyr)) ranged from 0.46 to 0.92, 0.01 to 1.15, and 0.29 to 1.15, respectively, reflecting shifts in both the sources and intensities of PAH inputs. These ratios are essential for distinguishing pyrogenic (biomass burning) from petrogenic origins. The observed diagnostic ratios indicate that biomass burning predominantly contributed to PAH emissions during periods of intensified fire activity (Fig. 5).

The n-alkane ratio (C25 + C27 + C29)/(C29 + C31) serves as a reliable biomarker for reconstructing past vegetation dynamics and inferring paleoclimatic conditions. This ratio reflects the relative contributions of woody to herbaceous plants, providing insights into terrestrial higher plant inputs. An elevated grass/wood ratio indicates a relative increase in the contribution of woody plants compared to herbaceous vegetation. In the CX profile, this ratio varied from 0.94 to 3.26 over the past 5000 years, with an average of 1.63 ± 0.46. The significant fluctuations between 3000 and 1000 year BP may be attributed to changes in vegetation composition driven by climatic variability and human activities (Fig. 3)^68,69^.

## Discussion

### The links between PAHs, fire and black carbon in sediments

Charcoal and black carbon (BC), including char and soot, are widely used as proxies for reconstructing paleo-fire events^7,8^. PAHs, recognized as reliable proxies, are particularly valuable for reconstructing paleo-fire intensity^7,41–43,70^. However, charcoal and BC exhibit spatial and temporal limitations in paleo-fire reconstructions^71^. The integration of multiple fire proxies can significantly enhance our understanding of fire regimes^14,28,42,43^.

Correlation statistical analysis indicate there was a correlation between char and low molecular weight PAHs (3-ring PAHs) fluxes (Table 3; *R* = 0.57 *P* < 0.01), while there was a significant correlation between char and HMW PAHs fluxes in the CX site (Table 3; *R* = 0.75 *P* < 0.01). these findings suggest that the combustion sources of char and HMW PAHs in the CX sediments are both homogeneous and primarily pyrogenic in origin. These results are consistent with previous research conducted on the southern Loess Plateau during the prehistoric period^72,73^.

**Table 3:** Correlation analysis of Char, soot, charcoal (< 50 μm), charcoal (> 100 μm), low molecular weight PAHs (2-ring PAHs) and HMW PAHs from the CX profile.

	Char	Soot	Charcoal (<50 μm)	Charcoal (>100 μm)	LWM PAHs	HWM PAHs
Char	1	0.77**	0.51**	0.54**	0.57**	0.75**
Soot		1	0.59**	0.56**	0.48**	0.60**
Charcoal (<50 μm)			1	0.82**	-0.061	0.48**
Charcoal (>100 μm)				1	0.11	0.29
LWM PAHs					1	0.76**
HWM PAHs						1

*Indicating significant correlation (< 0.05).

**Indicating significant correlation (< 0.01).

However, the non-synchronous peaks of black carbon (char) and PAHs observed in the study profile may be attributed to the varying components of pyrogenic derivatives, which are influenced by the combustion temperatures^17,45,74^. Previous fuel combustion experiments have shown that char combustion occurs at temperatures above 350 °C, while soot combustion takes place at over 700 °C. Low molecular weight PAHs (3-ring PAHs), such as naphthalene, typically combustion occurs between 300 °C and 400 °C, whereas HMW PAHs combustion occurs between 500 °C and 600 °C with granular form^75,76^.

Moreover, these asynchronous peaks also reflect differences in varying source area distances and transport mechanisms^74^. For example, PAH fluxes and charcoal deposition rates from central California and Yosemite National Park, Swamp Lake, suggest a correlation between low and medium molecular weight PAH fluxes and macro-charcoal (> 250 μm). This implies that they are primarily derived from at local sources. However, the small sample size of just 10 samples limits the robustness of statistical testing in the study site^66^. Compared the abundance of PAHs in 52 late Holocene peat samples from Borneo with those of two size fractions of charcoal particles. Their findings revealed that the depth distribution (particles/cm³) of high-molecular-weight PAHs closely matched that of the charcoal particles in both the > 250 μm and 125–250 μm size ranges, suggesting that these compounds originated from local sources^77^. Additionally, the asynchronous behavior is linked to the differing physicochemical properties of fire events^17,45,74^. Previous research has demonstrated that volatile low-molecular-weight PAHs tend to be deposited farther from their combustion source, while particulate high-molecular-weight PAHs typically settle closer to the source^78,79^. In soil environments, low-molecular-weight PAHs (2-ring PAHs) are more likely to interact with humus and undergo microbial degradation, whereas high-molecular-weight PAHs are closely associated with char, originating from black carbon aerosols produced by natural wildfires, land clearing, agricultural straw burning, and fossil fuel combustion^80^. PAHs and micro-charcoal levels in sediments from the Jinluojia archaeological site in China indicate two significant periods of large-scale tree exploitation for fuel during the Eastern Zhou Dynasty and earlier societies^81,82^. This corresponds to population growth, increased iron production, and frequent warfare, all of which contributed to intensified forest burning, particularly during wartime^81,82^. Similarly, low-molecular-weight PAHs (3-ring PAHs) concentrations at the Yangguanzhai archaeological site in the southern Loess Plateau, peaking in the mid-Holocene, suggest increased anthropogenic biomass burning. The presence of pyrogenic PAHs, particularly low-molecular-weight compounds (3-ring PAHs), points to fuelwood and woody plant combustion^73^. In contrast, high-molecular-weight PAHs (4-6-ring PAHs) are less abundant, as they are typically produced at higher temperatures (e.g., fossil fuel combustion) and are associated with coarse particles that do not travel far from their source^43^.

In conclusion, this study distinguishes two distinct types of anthropogenic biomass burning during prehistoric periods: low-temperature smoldering fires, likely driven by deforestation for land clearing, and high-temperature flaming fires, potentially linked to warfare. The identification of these fire pattern provides valuable insights into the evolution of fire regime, human activities, and their interactions with climate change in the region.

### Source of PAHs

The diversity of PAHs sources, sedimentary environments, and transport mechanisms presents challenges for evaluating the overall distribution of PAHs. In this context, diagnostic PAH ratios may more effectively reflect shifts in fire regimes than overall concentration data, allowing for better differentiation of source contributions^83^. Previous studies have demonstrated that the diagnostic ratios of PAHs with an An/(An + Phe) ratio > 0.10 are useful for indicating pyrogenic sources^83^. This ratio was detected for most samples of PAHs (Fig. 5). The IcdP/(IcdP + Bghi) ratios were associated with biomass burning. When ratios of IcdP/(IcdP + Bghi) > 0.5, this suggests that PAHs were generally from wood or coal combustion, ratio values of 0.20–0.50 suggests liquid fossil fuel combustion, and a low ratio (< 0.20) source (Yunker et al., 2002) (Fig. 5). Most samples with IcdP/(IcdP + Bghi) ratios > 0.50 could have all been from wood biomass burning sources. An Flu /(Flu + Pyr) ratios > 0.50 from most samples suggest that PAHs may be from wood combustion. In summary, all of these ratios (An /(An + Phe), Flu /(Flu + Pyr) and IcdP/(IcdP + BghiP)) suggest that most PAHs components of all samples may be derived from pyrogenic sources and biomass burning^83^.

**Fig. 7:**
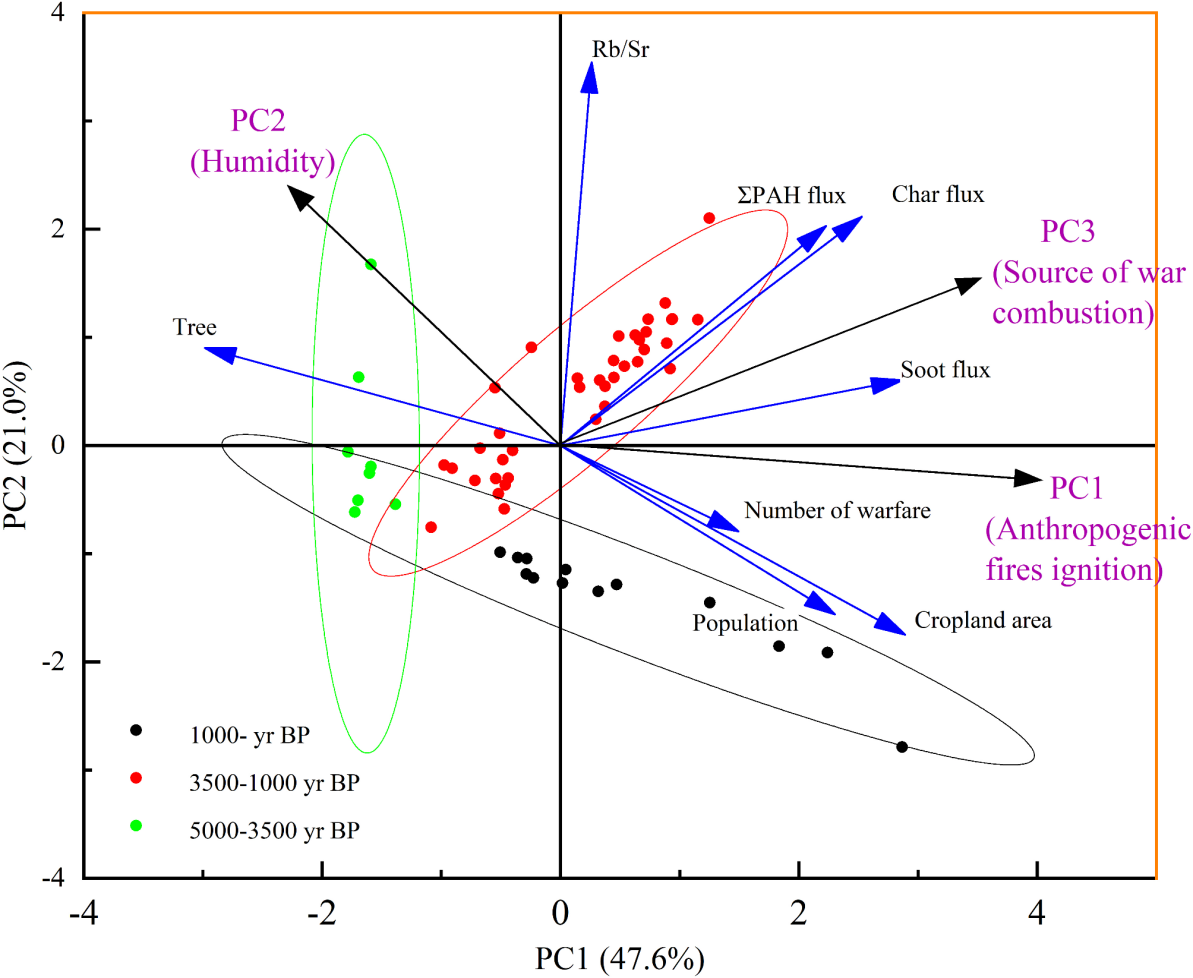
Principal component analysis (PCA) matrix of fire-related factors from the Changxu (CX) profiles during the Holocene. For the CX profile, PC1 corresponds to anthropogenic fire ignition, PC2 reflects humidity, and PC3 indicates fire sources related to warfare. Yellow, red, and black data points represent samples from 5000–3500 year BP, 3500–1000 year BP, and 1000 year BP to the present, respectively.

Principal component analysis (PCA) was conducted to identify the primary factors shaping fire activity over the past 5000 years, explaining 81.1% of the total variance through three principal components (PC1, PC2, and PC3; Table 4; Fig. 7). The robustness of the dataset was confirmed using the Kaiser-Meyer-Olkin (KMO) measure of sampling adequacy (0.70) and Bartlett’s test of sphericity, indicating that the data were suitable for factor analysis^84,85^.

**Table 4:** Principal component analysis loadings of Fire-Related factors from the CX profile.

Element	Principle component (PC)		
	PC1	PC2	PC3
Char flux	**0.755**	0.563	0.062
Soot flux	**0.874**	0.241	−0.139
∑PAHs	0.028	0.088	**0.781**
Tree	−0.862	0.327	0.095
Rb/Sr	0.477	**0.804**	−0.06
Population	**0.654**	−0.454	−0.328
Number of warfare	0.416	−0.23	**0.634**
Cropland area	**0.85**	−0.408	0.135
Total variance (%)	47.576	20.962	12.54
Cumulative (%)	47.576	68.539	81.079
Estimated source	Anthropogenic fires ignition	humidity	source of war combustion

Significant values are in bold.

PC1 accounted for 47.58% of the total variance, primarily composed of char flux, soot flux, population density, and cropland area, with respective eigenvalues of 0.76, 0.87, 0.65, and 0.85. Char and soot fluxes, representing direct proxies for fire intensity, reflect biomass burning processes such as slash-and-burn agriculture and forest clearing, while population density and cropland expansion highlight human-driven land-use changes^86,87^. Archaeological records show that the ancient people of the Longshan culture began to open up land for agriculture and strengthen pottery making and smelting activities during 5000 − 4000 years BP^88^. A pollen charcoal record from the Tuoji Island and Daqin Island sites in Changdao, Shandong Province shows that the disappearance of the island’s forests is closely related to the existence of a smelting site^89^. 4500 − 3000 years ago, due to the use of knives and fire by people, the limited forests were cut down. It can be seen that the Changdao case reflects that the human damage to the forest at that time was quite serious. According to historical records, the Qing Dynasty (1636–1912 CE) was a period of rapid population growth in Shandong, which increased to more than 25 million in the 32nd year of Qianlong (1767 CE)^90^. PC1 was identified as Anthropogenic fires ignition.

PC2 constituted 20.96% of the total variance and consisted of the Rb/Sr ratio (with eigenvalue 0.80, respectively), a well-established proxy for climatic conditions, particularly humidity and weathering intensity^91,92^. Higher Rb/Sr values suggest wetter climates, where increased moisture content in vegetation reduces flammability, thereby suppressing fire activity. The peak occurred during 4200–3800 years BP, which is consistent with geochemical trends and many evidences from climate proxy indicators in the monsoon region of China, indicating that a climate abrupt change event occurred during this period^93,94^, with a significant cooling event, cold and dry climate changes in the region, and enhanced extreme precipitation, multiple floods, forced people to migrate from the lower alluvial plains to the higher terraces, and a reduction in population and biomass burning during the decline of civilization in the late Neolithic^95,96^. PC2 was identified as humidity.

PC3 accounted for 12.54% of the total variance and was primarily influenced by ∑PAHs flux and warfare (eigenvalues of 0.78 and 0.63, respectively). PAHs serve as molecular indicators of biomass burning and fossil fuel combustion, while warfare events reflect anthropogenic fires triggered by deforestation and resource destruction during conflicts. As shown by the yellow line in Fig. 3q, PC3 exhibited marked increases during periods of intensified warfare, such as late Eastern Han Dynasty (25–220 CE). Historical records highlight the environmental consequences of large-scale military campaigns, where fire was employed as both a weapon and a consequence of resource exploitation. A notable example is the Battle of Bowangpo (202 CE), which led to widespread deforestation during the Eastern Han Dynasty^97^. PC3 was identified as source of war combustion.

The results reveal that fire regimes in the study area were driven by a complex interplay of anthropogenic influences, climatic variability, and war.

### Climate, fire pattern and human activity(warfare)

The sedimentary fire history from the CX profile records a nonlinear intrinsic coupling among the variation of black carbon and PAH fluxes, climate changes, and transformations in cultural settlements and land use over the past 5,000 years (Fig. 5). The transitions between these two burning modes are closely intertwined with climate variability and shifts in the intensity of human activities. Furthermore, they reflect the evolving patterns of human interaction with the environment and the feedback mechanisms of anthropogenic activities in response to the East Asian Monsoon climate.

#### Infrequent wildfires occurrence during the middle and late of holocene (5000 − 3500 year BP.)

The oxygen isotope record from the Shandong Cave speleothem, alongside δ^18^O data from ice cores^98,99^ (Fig. 3), reveals that the mid-Holocene climate in this region was warmer and wetter than it is today^100^. Pollen and n-alkane proxies reveal a vegetation shift from mixed broad-leaved coniferous forests to a landscape dominated by mixed broad-leaved forests and grassland(an increasing in *Chenopodium* and *Artemisia* taxa)^100^. Char and PAH fluxes suggest generally low wildfire occurrence during this period. The occurrence and spread of large-scale wildfires may be inhibited by the warm and humid climate conditions (PC2) at this time. However, a distinct peak in total PAH and char fluxes indicate a marked increase biomass burning between 5000 and 4500 year BP (Fig. 3). so that the production of black carbon in the region is limited to local inputs (Fig. 7)^101^. PAH characteristic ratio analysis indicates that source of PAHs was the primary biomass combustion during this period (Fig. 5). Archaeological evidence reveals minimal human activity in the region^101^. The charcoal record indicates a limited fire occurrence. specifically linked to a short-lived dry and cold event during 5200 year BP. (Fig. 3)^102^. Additionally, a decline in fire activity around 4500–4000 years BP correlates with a brief “deterioration” event (4200–4000 years BP) during the mid-Holocene, which was characterized by frequent droughts and floods associated with a weakened summer monsoon (Fig. 3)^103^. Climatic proxies from China’s monsoonal regions indicate an abrupt climatic event occurring between 4200 and 4000 year BP^104–106^. Thus, the peak in wildfire observed around 5000–4500 year BP in this study region appears primarily driven by natural factors. This period of climatic deterioration, marked by severe drought, disrupted dryland agriculture, causing food shortages and population migrations. As a result, the Longshan Culture was replaced by the Yue Shi Culture^89,107^. It is evident that climatic anomalies between 5000 and 3500 year BP. played a key role in the occurrence of wildfires.

#### High frequency anthropogenic fires during the late of holocene (3500 − 1000 year BP)

Distinguishing human-ignited fires from agriculture-driven wildfires based solely on fire proxies presents a significant challenge in paleofire research. To overcome this limitation, we propose an integrative approach that combines diverse fire regime reconstructions with paleoclimate and paleoecological records. By applying multivariate regression and principal component analysis, we aim to disentangle the primary drivers of anthropogenic fire events, determining whether they originated from agricultural practices or warfare. This comprehensive framework will provide novel insights into human-environment interactions and the socioecological impacts of fire, advancing beyond the scope of previous studies.

Between 3500 and 1000 year BP, the study region experienced a climatic transition from warm and humid to cooler and drier conditions, characterized by increased seasonal precipitation variability^100^. This shift is recoded by oxygen isotopic records from Shandong Cave speleothems and δ^18^O data from ice cores^98,99^ (Fig. 3). Pollen records and n-alkane proxies indicate that during this period, the region vegetation was a forest-grassland landscape, with mixed coniferous and broad-leaved forests alongside grasslands dominated by *Chenopodium* and *Artemisia* taxa^100^. Episodic fire activity increases were detected at the study site during distinct intervals: 3500–3000 year BP, 2500–2400 year BP, 2000–1800 year BP, and 1800–1400 year BP. These intervals correspond to the major historical periods in China, including the Shang Dynasty, Warring States period, Eastern Han Dynasty, and the Wei, Jin, and Northern and Southern Dynasties (Fig. 3). Principal component analysis indicates that anthropogenic biomass combustion (PC1) and war-related combustion (PC3) were the primary factor influencing fire occurrence during distinct intervals **(**nearly 60% of the variance; Fig. 7**).** Archaeobotanical evidence from Shang Dynasty sites confirms that rice cultivation was widespread in Shandong, facilitated by the region’s warm and humid hydrothermal conditions^108^. These findings suggest that the marked increase in black carbon(char) flux during the Shang Dynasty resulted from biomass combustion linked to agricultural practices, alongside fluvial erosion and redeposition of charred material. Forest cover during this period is estimated to have remained relatively high, at approximately 46%^90^. The peaks in biomass burning recorded in the CX profile during between 3500 and 3000 year BP could be anthropogenic in origin, as indicated by elevated levels of black carbon and low-molecular-weight PAHs (3-ring PAHs) (Fig. 3). Meanwhile, the nature landscape in the study region has shifted from fire-resistant primary forests to flammable secondary shrublands due to extensive deforestation and land cultivation during those periods. Once persistent drought weather occurs, this highly flammable shrubland becomes particularly prone to smoldering fires, posing a significant risk for future forest fire outbreaks. This pattern resembles the situation observed in the Greater Khingan Mountains of China, where prolonged summer droughts have significantly reduced the moisture content of dead branches and leaves, creating favorable conditions for smoldering fire ignition^109^.

**Fig. 6:**
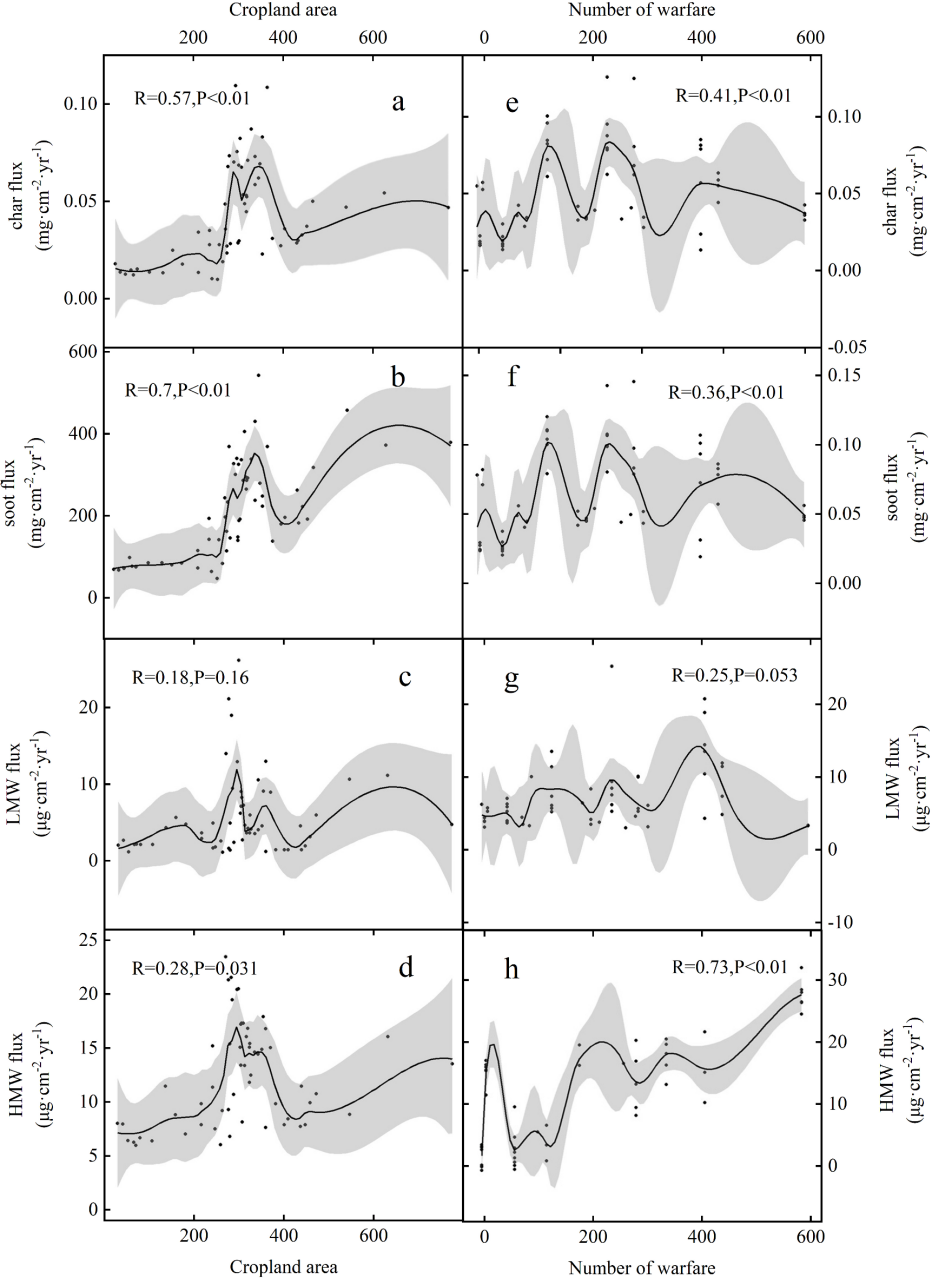
Correlation between char flux, soot flux, low-molecular-weight (2-ring) PAHs, HMW PAHs, cropland area expansion, and warfare events.

In contrast, peaks in the flux of high-molecular-weight (HMW) PAHs were identified at the study site during three distinct periods: between 2500 and 2400 year BP, between 2000 and 1800 year BP, and between 1800 and 1400 year BP. These episodic increases in fire activity coincide with major periods of conflict in Chinese history, specifically the Warring States period, the Eastern Han Dynasty, and the Wei, Jin, Northern, and Southern Dynasties. Correlation analyses reveal a strong positive correlation between HMW PAHs and the number of wars in the study region between 3000 and 1000 year BP (*R* = 0.73, *P* < 0.01; Fig. 6). In contrast, the correlation between char and soot fluxes and the number of wars is comparatively weaker (*R* = 0.41, *P* < 0.01 and *R* = 0.36, *P* < 0.01; Fig. 6). No significant correlation was observed between HMW PAHs and the cropland area during this period (*R* = 0.28, *P* = 0.031; Fig. 6). However, char and soot fluxes showed a strong correlation with the cropland area (*R* = 0.57, *P* < 0.01 and *R* = 0.70, *P* < 0.01, respectively; Fig. 6). Modern fuel combustion experiments indicate that peaks in the flux of HMW PAHs correspond to significant increases in high-intensity local pyrogenic biomass burning during these times^110^. The characteristic ratios of PAH components further confirm that biomass combustion was the primary source of these emissions (Fig. 5). Principal component analysis (PCA) reveals that anthropogenic biomass combustion (PC1) and war-related combustion (PC3) were the primary factors influencing fire occurrence during these periods, accounting for nearly 60% of the variance (Fig. 7).

Historical records indicate that the number and frequency of wars during the Warring States period, the Eastern Han Dynasty, and the Wei, Jin, and Northern and Southern Dynasties were 661 (1.2 wars per year), 682 (1.6 wars per year), and 1,677 (4.6 wars per year), respectively. The Northern and Southern Dynasties (420–589 CE) represent one of the most turbulent periods in ancient Chinese history, marked by frequent warfare and political instability. Over the course of 169 years, this period witnessed more than a hundred wars and military conflicts. The study area, a key strategic region in northern China, experienced some of the most intense and persistent military activities of the time^111,112^.

Fire was often used as a military tactic, and logging for military purposes significantly impacted forest resources during this period of frequent conflict and numerous vassal states^113,114^. From the Cao Wei and Jin dynasties to the Northern and Southern Dynasties, the forests surrounding the study region were extensively deforested. The use of fire in military campaigns, such as the Battle of Bowangpo and Lu Xun’s burning of Liu Bei’s camp at Yiling, resulted in large-scale deforestation along a 700-mile stretch. During the Southern Dynasties, Emperor Wu of Liang mobilized 200,000 soldiers to construct the Fushan Dam using timber and stone-filled frames to divert the Huai River and flood the Northern Wei city of Shouyang, further contributing to deforestation^115^. Conversely, the population decline following the War of the Eight Princes and subsequent conflicts during the Northern and Southern Dynasties led to land abandonment in the lower and middle reaches of the Huai River, which, in turn, promoted vegetation recovery^97^. The correlation between HMW PAHs and warfare suggests that the intense biomass burning events during these periods were closely linked to recurring conflicts in the study region (Fig. 6).

We note that these observations strongly suggest an inherent causal relationship between variations in high-ring PAHs, the incidence of warfare, vegetation dynamics, and climate anomalies in the past of 3000 year.

**Fig. 8:**
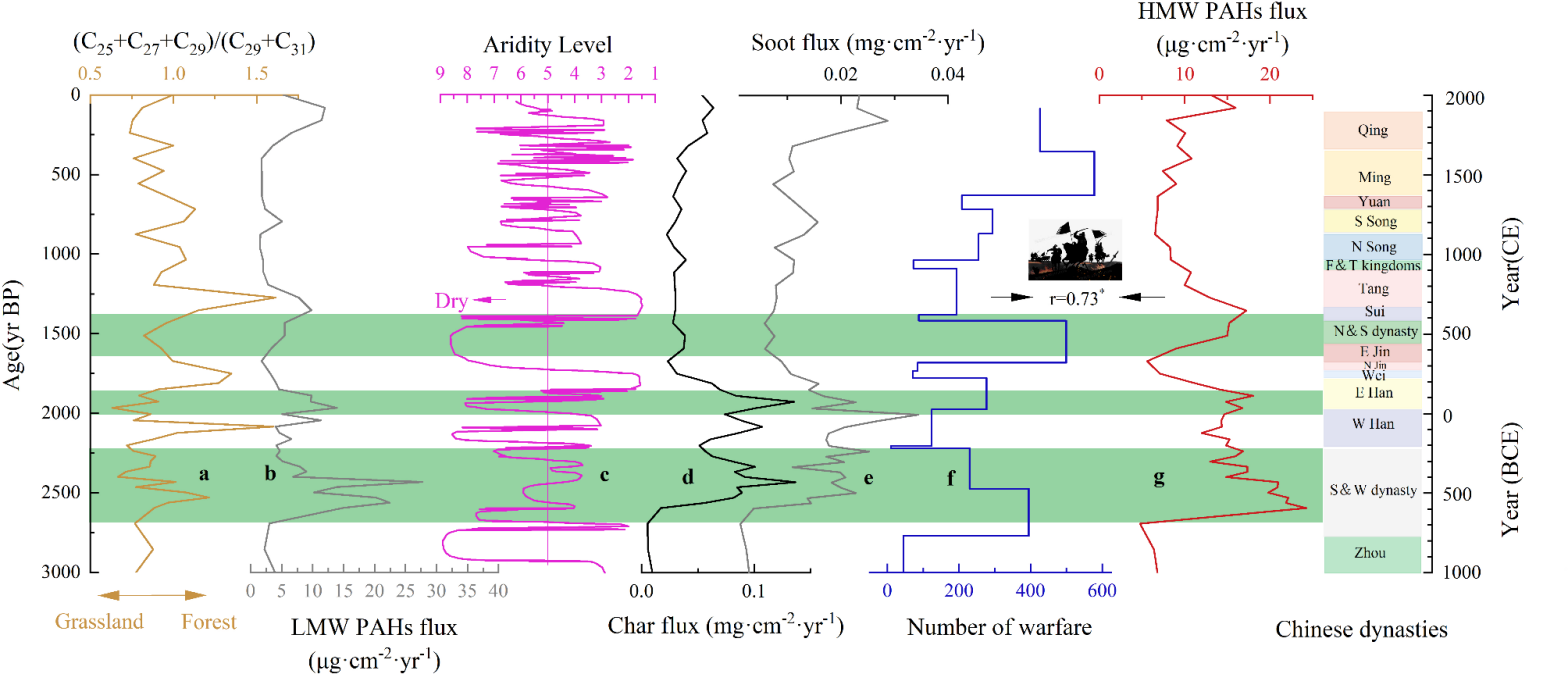
Comparison of paleofire history with human activities and paleo-climatic records over the past 3000 years BP. (**a**) Ratio of (C25 + C27 + C29) to (C29 + C31) from the CX site. (**b**) Low molecular weight PAHs (2-ring PAHs) flux trends from the CX site. (**c**) Aridity index for eastern China^128^. (**d**) Trends in char and soot flux from the CX site. (**e**) Number of warfare events in eastern China^111^. (**f**) HMW PAHs flux trends from the CX site.

Climate proxies suggest that the region experienced warmer and wetter conditions during the Qin-Han period (2200–2000 year BP) and the Sui-Tang period (1400–1100 year BP), with mean annual temperatures elevated by 1–2 °C and precipitation levels 200–300 mm higher than present^116,117^. The increased ratio of (C25 + C27 + C29)/(C29 + C31) indicates a slight rise in the proportion of woody vegetation in forest composition, driven by warming temperatures. Concurrently, the fluxes of high-molecular-weight PAHs remained low, while low-molecular-weight PAH fluxes exhibited a stable level inferred by records of PAH during these periods in the study region (Fig. 8). Historical records indicate that millet, rice, and soybeans were extensively cultivated on the Shandong Peninsula during these periods^118^. During the Qin-Han period (2200–2000 year BP) and the Medieval Warm Period (Sui-Tang period), favorable thermal and hydrological conditions, driven by an intensified East Asian monsoon, supported agricultural expansion, population growth, and social stability. These optimal conditions likely contributed to a decline in high-molecular-weight PAH fluxes, while low-molecular-weight PAH fluxes remained widespread and relatively stable^116,117^.

Conversely, Climate proxies indicate the region was colder and drier during the Eastern Han (2000–1800 year BP), and the Wei, Jin, and Northern and Southern Dynasties (1800–1400 year BP) (Fig. 8). and the late Ming to early Qing Dynasties (472–340 year BP, Little Ice Age)^116,119^. Paleoclimate records reveal that the region underwent abrupt cooling, arid conditions, and frequent extreme events, such as floods and droughts, due to the weakening of the East Asian monsoon during those periods^116,117^. The decline in the ratio of (C25 + C27 + C29)/(C29 + C31) indicates that, as temperatures decreased during this period, the proportion of woody vegetation in forest cover diminished, while the relative abundance of shrubs and grasslands increased. Concurrently, PAH records reveal an elevated flux of high-molecular-weight PAHs, whereas the flux of low-molecular-weight PAHs remained stable level. Pollen records from Ningjin Bo and stalagmites from Shihua Cave (Beijing) indicate a transition from a warm, humid climate to a colder, drier one during the Wei-Jin and Northern and Southern Dynasties^120,121^. This climatic shift prompted an expansion of herbaceous cover across the North China Plain and the later appearance of conifers, including spruce. As agricultural productivity declined, large areas of farmland were abandoned in favor of pastoralism. The period was marked by prolonged instability, with over 100 conflicts spanning 169 years during the Northern and Southern Dynasties^112,119^. This led to population loss, abandonment of arable land, and the dominance of secondary grasslands and shrublands. Under these conditions, biomass burning associated with warfare significantly increased emissions of high-molecular-weight PAHs, despite the region’s relatively low population density.

In summary, the high frequency of anthropogenic fires in the Lubei Plain during the late Holocene (3500–1000 year BP) can be attributed to two distinct human-driven fire regimes and their interaction with climate changes. During warmer, more favorable climatic periods, low-temperature smoldering fires were primarily driven by widespread agricultural activities, while warfare became a significant driver of high-temperature flaming fires under colder, drier conditions. These fire regimes facilitated a gradual shift in the region’s vegetation from primary forests to secondary shrublands. Persistent droughts played a crucial role in sustaining both the fire regimes and vegetation succession.

#### Widspread and stable biomass burning in the past of 1000 year ago

Climate proxies, including negative oxygen isotopes from speleothems in caves and positive oxygen isotopes from deep-sea sediments, indicate that the climate began to shift toward cooler and drier conditions over the past 1,000 years^100^. Pollen records and n-alkanes proxies indicate that the forest grassland landscape with coniferous forest and grassland vegetation (*Chenopodium* and *Artemisia*) during this time^100^. The fluxes of char and PAHs suggest that biomass combustion remained relatively stable during this stage. However, notable peaks in char and PAH fluxes were observed around 300 years ago. Principal component analysis indicated that anthropogenic biomass burning (PC1) was the primary influencing factor for regional fire occurrence during this period **(**Fig. 7).

The characteristic ratio analysis of PAHs further corroborates that biomass combustion was the principal source of PAHs during these periods. Climatic conditions approximately 1,000 years ago were marked by relative aridity, with low vegetation cover in the floodplain sedimentary environments. During this time, irrigation systems and a dry farming landscape had already been established in the study region. Anthropogenic biomass burning still remain a relative higher and stable level. Historical records indicate that by the 32nd year of Qianlong’s reign in the Qing Dynasty (1767), the population of Shandong had exceeded 25 million^122^. This rapid population growth drove extensive deforestation and intensified anthropogenic fire activity, leading to reduced vegetation cover and accelerated soil erosion.

## Conclusion

The PAHs preserved in the sediments of the Changxu (CX) section offer a comprehensive record of biomass burning patterns on the Lubei Plain over the past 5,000 years. Between 5000 and 3500 year BP, fire activity remained generally low. However, a pronounced peak in total PAH and charcoal fluxes between 5000 and 4500 year BP suggests a significant increase in regional burning, likely triggered by a short-lived cold and dry event around 5000 year BP. In contrast, the late Holocene (3500–1000 year BP) experienced a marked rise in anthropogenic fires driven by two distinct human-induced fire regimes and their interactions with climate variability. Low-temperature smoldering fires, largely resulting from extensive deforestation for agricultural expansion, whereas high-temperature flaming fires were primarily linked to warfare and conflict during this period. They played a critical role in reducing forest cover and accelerating soil erosion. Persistent drought conditions further amplified both fire regimes, creating conditions conducive to sustained burning. Over the past 1,000 years, the frequency, intensity, and spatial extent of biomass burning have remained relatively high and stable, reflecting continued human influence on fire regimes. Variations in high-molecular-weight (HMW) PAH flux provide a robust indicator of high-intensity pyrogenic combustion associated with warfare, highlighting the complex interplay between fire activity, societal dynamics, and land-use practices in response to climatic shifts.

The fire history of the Lubei Plain reveals intrinsic links between HMW PAHs, black carbon flux, warfare frequency, vegetation changes, and climate anomalies. Low-temperature smoldering fires are predominantly associated with prolonged anthropogenic biomass burning for agriculture under warm climatic conditions. In contrast, short-duration, high-temperature fires correspond to periods of social unrest and warfare, occurring across both warm and cold phases. However, further research is needed to fully understand the periodicity, frequency, intensity, and driving factors of these distinct fire patterns.

Integrating molecular biomarkers such as PAHs with black carbon and charcoal records, combined with principal component analysis and multivariate regression, provides a powerful framework for reconstructing past fire regimes and their feedbacks to climate variability. This comprehensive approach enhances our understanding of fire dynamics and the long-term interactions between human activity, climate change, and environmental transformation.

## Electronic supplementary material

Below is the link to the electronic supplementary material.

Supplementary Material 1

## Data Availability

Data is provided within supplementary information files.
