# Author Correction: Record of polycyclic aromatic hydrocarbons (PAHs) from prehistoric sediments and human activity in the Lubei plain of China

**DOI:** 10.1038/s41598-025-99044-2

**Published:** 2025-05-08

**Authors:** Huanrong Zuo, Zhihai Tan, Yongming M. Han, Longjiang Mao, Shuxin Zheng, Qi Zhang, Meng Wang, Shihao Li

**Affiliations:** 1 School of Environment and Chemistry Engineering, Xi’an Polytechnic University, Xi’an , 710048 Shaanxi China; 2 State Key Laboratory of Loess and Quaternary Geology, Key Laboratory of Aerosol Chemistry and Physics, Institute of Earth Environment, Chinese Academy of Sciences, Xi’an, 710061 Shaanxi China; 3 School of Marine Sciences, Nanjing University of Information Science & Technology, Nanjing, 210044 Jiangsu China

Correction to: *Scientific Reports* 10.1038/s41598-025-91885-1, published online 01 March 2025

The acknowledgement section in the original version of this Article was omitted. The Acknowledgement section now reads:

This research is sponsored by grants from National Natural Science Foundation of China (No. 42373085), and is also supported by the Strategic Priority Research Program of Chinese Academy of Sciences (Grant No. XDB 40000000); Natural Science Basic Research Program of Shaanxi Province (No. 2023-JC-YB-226); Project Supported by the Foundation for Innovative Research Groups of the National Natural Science Foundation of China (No. 42221003). Fund of the State Key Laboratory of Loess and Quaternary Geology Chinese Academy of Sciences (No. SKLLQG 2223); We gratefully acknowledge the helpful and valuable comments provided by the editor and the anonymous reviewers, which greatly could improve the manuscript.

The original Article has been corrected.

